# Respiratory distress syndrome management in resource limited settings—Current evidence and opportunities in 2022

**DOI:** 10.3389/fped.2022.961509

**Published:** 2022-07-29

**Authors:** Osayame A. Ekhaguere, Ikechukwu R. Okonkwo, Maneesh Batra, Anna B. Hedstrom

**Affiliations:** ^1^Department of Pediatrics, Indiana University School of Medicine, Indianapolis, IN, United States; ^2^Department of Pediatrics, University of Benin Teaching Hospital, Benin City, Nigeria; ^3^Departments of Pediatrics and Global Health, University of Washington, Seattle, WA, United States

**Keywords:** respiratory distress syndrome (RDS), low- and middle-income countries, treatment, surfactant, continuous positive airway pressure (CPAP), low resource, prematurity

## Abstract

The complications of prematurity are the leading cause of neonatal mortality worldwide, with the highest burden in the low- and middle-income countries of South Asia and Sub-Saharan Africa. A major driver of this prematurity-related neonatal mortality is respiratory distress syndrome due to immature lungs and surfactant deficiency. The World Health Organization's Every Newborn Action Plan target is for 80% of districts to have resources available to care for small and sick newborns, including premature infants with respiratory distress syndrome. Evidence-based interventions for respiratory distress syndrome management exist for the peripartum, delivery and neonatal intensive care period- however, cost, resources, and infrastructure limit their availability in low- and middle-income countries. Existing research and implementation gaps include the safe use of antenatal corticosteroid in non-tertiary settings, establishing emergency transportation services from low to high level care facilities, optimized delivery room resuscitation, provision of affordable caffeine and surfactant as well as implementing non-traditional methods of surfactant administration. There is also a need to optimize affordable continuous positive airway pressure devices able to blend oxygen, provide humidity and deliver reliable pressure. If the high prematurity-related neonatal mortality experienced in low- and middle-income countries is to be mitigated, a concerted effort by researchers, implementers and policy developers is required to address these key modalities.

## Background

Childhood mortality is predominantly driven by deaths in the neonatal period (the first 28 days of life) (1). In the last 30 years, reductions in neonatal mortality have not kept pace with those beyond the first month of life (1). Consequently, 47% or an estimated 2.4 million of all childhood deaths occur in the newborn period, with births occurring before 32 weeks gestation carrying the highest risk of death (2, 3). To focus the world's attention on needed improvements to close this gap, the Sustainable Development Goals revised targets in 2015 to reduce neonatal mortality to 12 per 1,000 live births by 2030 (4). The Every Newborn Action Plan (ENAP) identified the management of the complications of prematurity as a high-yield area for improvement critical to reducing neonatal deaths (2, 5).

**Figure 1:**
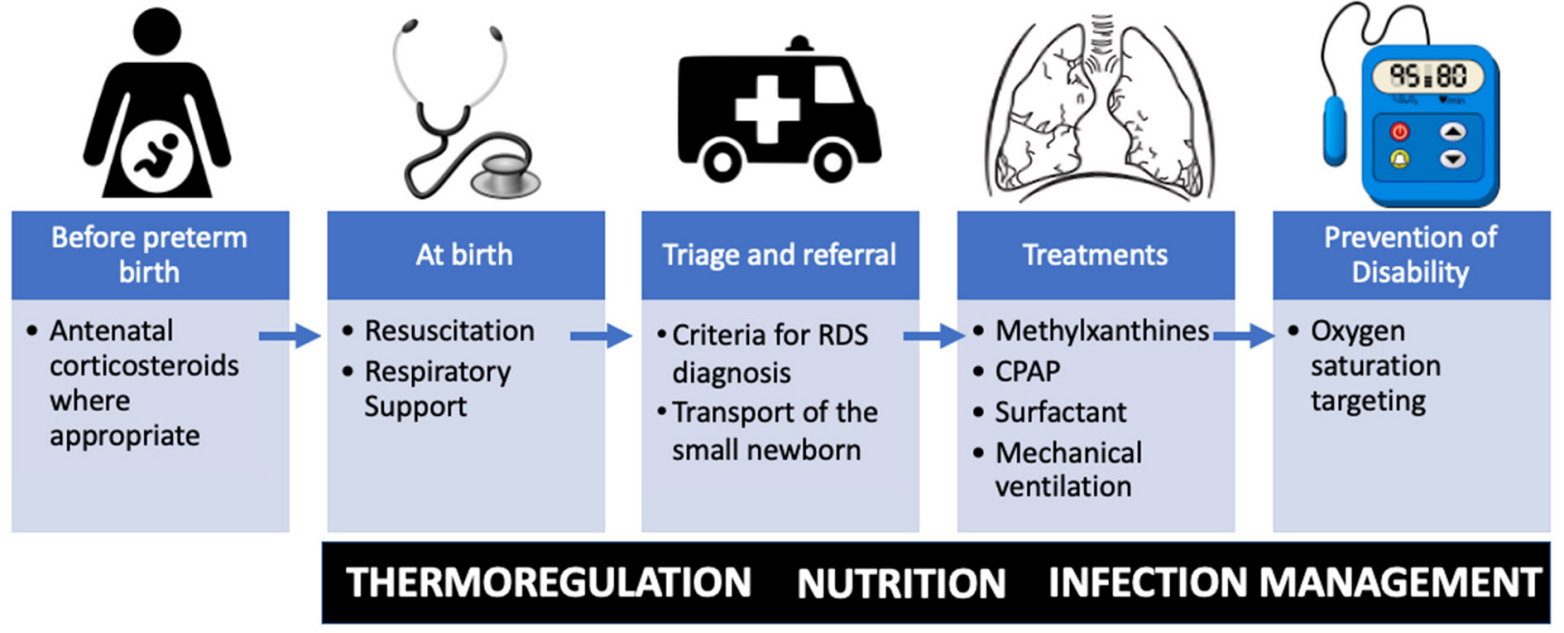
Essential therapies for respiratory distress syndrome (RDS) in resource limited settings along the time course of preterm birth. Important fundamental premature care includes thermoregulation, nutrition, and management of infection. CPAP, continuous positive airway pressure.

Between and within-country variation in premature birth rates and prematurity-related mortality exist, with low- and middle-income countries (LMIC) carrying the highest burden (6). Over 90% of extremely preterm babies (<28 weeks) born in LMICs die within the first few days of life, while <10% of extremely preterm babies die in high-income countries (HICs) (6). Improving access to facilities capable of delivering quality neonatal care for small and sick newborns has been identified as a target of the ENAP (5). Specifically, the ENAP coverage target has called out the goal of having 80% of districts with available care for small and sick newborns (7). World Health Organization (WHO) guidelines on transforming care for small and sick newborns target key treatment modalities (Figure 1) for primary health facilities including neonatal resuscitation; secondary facilities including oxygen, continuous positive airway pressure and methylxanthines; and in addition to these, for tertiary facilities surfactant and mechanical ventilation (8).

The drivers of prematurity related mortality are multifaceted and related to the immature organ-systems of the preterm newborn. However, respiratory distress syndrome (RDS), which results from lung immaturity and surfactant deficiency, contributes about 45% of case-fatality due to prematurity in LMICs (9). Risk of RDS associated mortality is inversely related to the degree of prematurity. Given its contribution to prematurity-specific mortality, optimizing and scaling RDS specific interventions is paramount for reducing neonatal mortality at a population level, as is targeted by ENAP. In this paper, we review the pathophysiology of RDS, review RDS-specific interventions including their mode of action, available evidence that supports their use in High and LMICs and discuss research and operational gaps that exist which are specific to LMICs.

### Respiratory distress syndrome

The development of RDS begins with impaired or delayed surfactant synthesis and secretion in the immature lung. Between 24 to 28 weeks of gestation, type II alveolar epithelial cells begin production of surfactant, however, this innate surfactant production is often insufficient for extra-uterine life until after 35 weeks (10). The primary function of surfactant is to reduce the surface tension of the air-liquid interface in the alveoli. When deficient, atelectasis, ventilation-perfusion mismatch, and hypoventilation ensues with resultant hypoxemia, hypercarbia, and impaired endothelial and epithelial integrity with leakage of proteinaceous exudate and formation of hyaline membranes and injury to the immature lung (11). Perinatal risk factors for developing RDS include lower gestational age and birth weight, male sex, cesarean delivery without labor, maternal diabetes, and perinatal hypoxic-ischemic events (11–13). In contrast, antenatal steroids, hypertensive disorders in pregnancy, and prolonged rupture of membranes are perinatal factors associated with a reduced risk of developing RDS (12, 14). Certain postnatal conditions including acidosis, hypothermia, hyperoxia, poor perfusion, baro and volutrauma from assisted ventilation, affect surfactant production, function, and metabolism (11).

The incidence of RDS is inversely related to gestational age. It occurs in 98% of preterm infants between 22 and 24 weeks gestation but only 25% of those with birth weight between 1,251 to 1,500 grams (15, 16). The signs of RDS are non-specific, and not all preterm infants below 34 weeks gestation presenting with respiratory distress have RDS. Examples of conditions that can mimic RDS include retained lung fluid, meconium aspiration syndrome, persistent pulmonary hypertension, and pulmonary hypoplasia (11, 17). RDS, however, clinically worsens in the first few days after birth hence, early diagnosis is important to ensure prompt treatment and transfer as necessary. RDS can increase the risk of pneumothorax and in severe cases, or where appropriate therapy is not available, can lead to respiratory failure and death (17).

### Diagnostic challenges

In HICs, RDS is diagnosed in preterm infants who have signs including supraclavicular, intercostal, and subcostal retraction, grunting and flaring of the nares, requirement for supplemental oxygen as dictated by hypoxia from pulse oximetry, and have chest radiographic findings of diffuse haziness and air bronchograms (18). Additionally, blood gas analysis to assess for acidosis, hypoxemia, and hypercapnia may be included in the diagnostic criteria. These parameters are also used to determine the need for the initiation or escalation of respiratory support.

**Figure 2:**
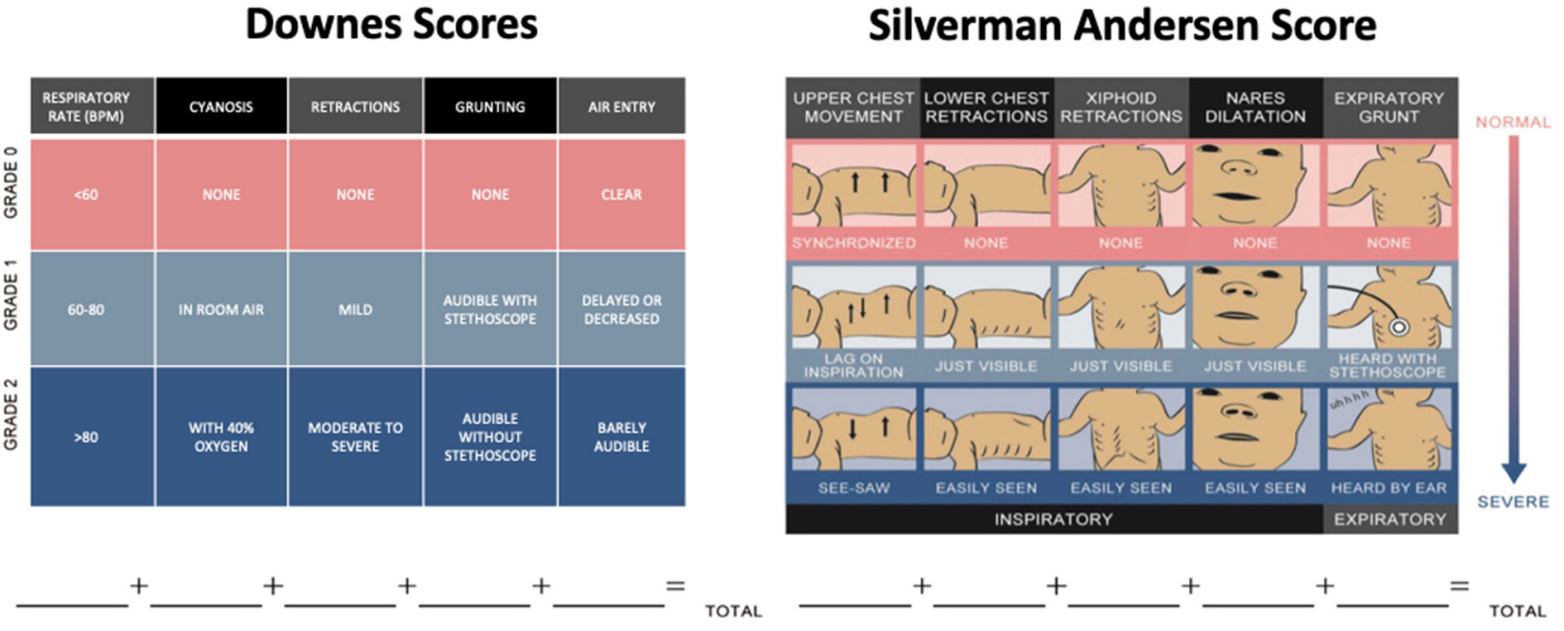
Scoring systems for respiratory distress syndrome. Downes and Silverman Andersen scores assign 0–2 points for each of five categories of respiratory distress (19, 20). Part of figure reprinted with permission from (25).

However, the use of chest radiographs, pulse oximetry and blood gas analysis are resource intensive and not commonly available in LRS. Hence, the most feasible assessments for RDS in LRS include objective criteria for assessing work of breathing, such as scoring systems that are simple, non-invasive, inexpensive and have shown both prognostic value and good inter-rater-reliability. Most commonly in use are the Downes score and the Silverman Andersen Respiratory Severity Score (19, 20). In one study among nurses trained to use the Silverman Andersen Score, the intra-class correlation coefficient was 0.88 (CI 0.72–0.98) (21). Similarly, in another study among nurses trained to use the Downes score reported an inter rater reliability of 0.71 (22). These scores assess work of breathing specific to the physiology of newborns such as chest wall flexibility and use of accessory respiratory muscles. Components of these scores include exam findings of work of breathing, cyanosis, and degree of tachypnea (Figure 2). Neonates are scored on each component from 0 to 2 and their total score is from 0 to 10, where 10 represents severe distress. Elevated scores correlate to an increased likelihood of requiring advanced respiratory support (23, 24). Silverman Andersen score in the 4–6 range is commonly used as a threshold for initiation, and titration of CPAP therapy given it can be serially repeated at the bedside as a type of vital sign (23, 25).

### Antenatal corticosteroids

In the 1960s, animal studies showed corticosteroids accelerated lung maturation (26). In subsequent human trials, antenatal corticosteroid (ACS) given to the pregnant woman prior to preterm birth improved survival in premature infants primarily by reducing RDS incidence (14). Consequently, ACS became the standard of care in pregnancies between 24 0/7 weeks and 33 6/7 weeks of gestation at risk of delivery within 7 days (27–29). In high-income settings, over 80% of at-risk pregnancies receive ACS (30, 31). However, its use in low resource settings remains low and sporadic (32). Betamethasone is most commonly used in HIC, however, it is not readily available in LRS (33). Hence, WHO has recommended the use of dexamethasone as a substitute to betamethasone (27). Evidence suggests both betamethasone and dexamethasone are equivalent in the effect (34, 35).

Informed by models and strong evidence from both high, and upper middle-income countries that suggested ACS may prevent prematurity-related neonatal mortality (36–38), the National Institutes of Child Health and Human Development sponsored the population-based ACS cluster-randomized trial (39). This trial was conducted in peri-urban and rural settings in geographical clusters in Argentina, Guatemala, India, Kenya, Pakistan and Zambia (39). Women assessed to have signs of preterm labor, preterm premature rupture of membranes, pre-eclampsia or eclampsia, or obstetric hemorrhage were included. The primary outcome was 28-day neonatal mortality among infants less than the 5th percentile for birthweight—a proxy for preterm birth as gestational age could not be reliably determined (39). The proportion of infants with birth weight <5th percentile was 5% (2,362/48,219) and 4% (2,094/51,523) in the intervention and control group, respectively. The results of this trial showed no benefit on mortality in neonates with birth weights <5th percentile, increased risk of mortality in neonates >2,500 g, and an increased incidence of maternal infection (39). Notable variations in the study outcome occurred by study region. Among African sites included in the trial, ACS was associated with increased risk of mortality among neonates with birth weights <5th percentile (39). Further sub-regional analysis to evaluate why better outcomes were observed in some regions and not others, suggested for example, that better obstetric and neonatal care may have been associated with the improved outcomes observed in Guatemala (40).

After this study, the WHO issued guarded recommendations for ACS to be used in health facilities capable of assessing gestational age, diagnosing maternal infection, and providing emergent obstetric and preterm neonatal care, including resuscitation, respiratory, thermal, and nutritional support (27). Subsequently, the Antenatal Corticosteroids for Improving Outcomes in preterm Newborns (ACTION-1) Trial-sponsored by the WHO-randomized pregnant women between 26 0/7 weeks and 33 6/7 weeks of gestation at centers capable of gestational age assessment, who were at risk for preterm birth to either dexamethasone or placebo. This trial showed ACS resulted in a 16% and 12% risk reduction in neonatal mortality and stillbirths (41). To evaluate the impact of ACS on late preterm infants or those >2,500 g, the ACTION-2 trial was conducted in pregnant women between 34 0/7 weeks and 36 6/7 weeks of gestation who were at risk of preterm labor (42). The results from the ACTION-2 trial, showed ACS did not increase the risk of mortality and reduced the need for resuscitation by 62% (42).

Taken together, these trials on ACS among pregnant women in LMICs at risk of preterm delivery indicate that with close monitoring and the provision of neonatal interventions for preterm newborns, ACS has the potential to substantially reduce prematurity-related mortality. However, a significant proportion of deliveries in LMICs still occur in resource limited settings where the quality of care required to reap the benefits of ACS may be unavailable. Research on how to effectively monitor women at risk of preterm delivery, optimize neonatal resuscitation and increase availability of level two newborn care including respiratory support with limited resources as recommended by WHO is required. Also, investments and research into optimizing the referral and safe transportation systems to higher-level facilities for the at-risk mother, would increase access to ACS.

#### Transport of the small and sick newborn

While desirable, *in utero* transfer of pregnancies at risk for preterm delivery to a center capable of providing high-quality obstetric and neonatal care is not always feasible (43, 44). WHO recommends small or sick newborns receive a timely referral through integrated newborn service pathways with continuity of care, including during transport (45). In high resource countries the practice of inter-healthcare facility transportation of critically ill neonates continues to expand and has evolved into mobile ICUs capable of delivering state-of-the-art critical care outside the NICU, thus maintaining or improving the continuum of care (46). Anticipating the need for transfer early, appropriate preparation for transfer, and ongoing high-quality care during transfer, are the cornerstones of quality neonatal transport systems (44). Maintaining early supportive care is especially important for the treatment of RDS including use of oxygen and CPAP. However, the availability and quality of referral systems in LMICs are limited (47). In a study from Nigeria that examined 411 neonatal transports, no referral information was available upon presentation to the tertiary referral center. In that study, only 4% arrived by ambulance, 0.7% in a transport incubator, and 7% were accompanied by a health professional (48). This pattern is common in most published observational studies from other countries as well (47, 49). Of the few studies from South Africa where medical services are more established, limitations in availability of resources and effective communication between facilities were limitations of the transport systems (46, 50).

Safe and timely inter-facility transport of small and sick newborn infants including those with RDS is critical to maintain the continuum of care from the referring to the referral hospital. National commitments, investments, and research into optimizing and examining the effect of inter-facility transport on neonatal mortality are critical for further improvements in prematurity-related mortality and morbidity.

#### Delivery room management

Initial respiratory support for some premature infants with RDS requires careful bag mask ventilation to establish lung functional residual capacity. In anticipation of a preterm delivery, appropriate preparation includes availability of a working self-inflating bag and appropriately sized facemask, as well as an emergently available provider trained in resuscitation to focus on the newborn. Helping Babies Breath is a low cost, well established program to train providers to perform these key interventions (5, 51, 52). Implementation of this program has been shown to reduce intrapartum-related stillbirths and 1-day neonatal mortality rate (53). Challenges for scale up of HBB, however, include limited time for training, retention of trained staff, and learners subsequently translating simulation skills into consistent behavioral change (54). Research suggests low dose, high frequency refresher strategies are associated with best retention (54). While the HBB program addresses resuscitation for the majority of births globally, the program is not targeted toward care of the preterm infant.

Tertiary facilities in LMIC may have greater capacity for resuscitation of the premature newborn than what is presented in HBB. Where resources allow, use of the Neonatal Resuscitation Program provides additional training for the initial management of preterm newborns, such as emergent endotracheal intubation and continuous positive airway pressure (CPAP) (55).

The Neonatal Resuscitation Program recommends the use of CPAP when respiratory distress persists after initial resuscitation despite establishment of a normal heart rate and spontaneous respiration (55). This use of CPAP shortly after delivery reduces the risk of subsequent intubation, surfactant use, and ventilator days and is a useful therapy where equipment and expertise are available. CPAP use among preterm infants does increase the risk of pneumothorax, so should be used with caution, and where availability of staff and resources to manage this complication are available (56–59).

#### Continuous positive airway pressure

The primary consequence of RDS (surfactant deficiency) is alveolar collapse and the loss of functional residual capacity. In spontaneously breathing infants with RDS, CPAP provides continuous distending pressure to the airway and lungs (45, 60). This pressure provides the driving force to overcome the elastic, flow-resistive, and inertial resistance of the respiratory system and restore functional residual capacity (61). The continuous pressure applied *via* a nasal interface enhances lung inflation, decreases work of breathing and is associated with decreased mortality due to RDS (60, 62). CPAP also decreases the risk of chronic lung disease, one of the major sequelae of RDS that often requires the baby receive pulmonary care up to or beyond term-corrected age (63).

CPAP devices can be grouped into two broad categories based on the method of pressure generation. Devices that use an adaptive flip valve located at the nasal interface to generate CPAP are termed variable flow devices (64). Devices that generate pressure by preventing gas egress from the circuit, because of an expiratory limb resistance or a titratable PEEP valve, are termed continuous flow devices (64). One form of continuous flow CPAP device is one where the expiratory limb is submerged in liquid with the depth of insertion coinciding with the pressure in the circuit. These “bubble CPAP” devices result in less failure of CPAP and can be generally made at a lower cost than other forms of CPAP (65). Most CPAP devices available in HIC are expensive, ranging from US$2000 to US$6000—not including the cost of consumables (66, 67).

Evidence from HIC suggest that compared to supplemental oxygen, the use of CPAP is an effective treatment for preterm infants with RDS (68). A 2020 Cochrane systematic review of CPAP vs. supplemental oxygen and the effect on treatment failure and death included five studies and 322 preterm infants with RDS from HICs. In this review, treatment with CPAP significantly lowered the risk of death, or use of mechanical ventilation [typical risk ratio (RR) 0.64, 95% confidence interval (CI) 0.50 to 0.82; typical risk difference (RD) −0.19, 95% CI −0.28 to −0.09] (68).

The cost of CPAP devices, unavailability of consumables and spare parts, maintenance needs, and dependence on electricity have limited the availability and use of commercialized CPAP devices in LMICs (67, 69). To fill this gap, multiple low-cost CPAP devices have emerged ranging from those crafted locally by providers at the bedside, to lower-cost commercialized devices (70–81). In comparison with commercial device available in HIC settings, the improvised devices lack some features raising questions regarding their efficacy and safety (72, 82–84). Specifically, the improvised devices often lack heated humidity relying only ambient humidity. The available tubing in low resource settings for assembly of CPAP are for standard nasal cannula which are of narrower bore than those typically used with commercialized CPAP circuits (85). The nasal cannula interface used are also not specifically designed to transmit pressure from CPAP and may attenuate the delivered pressure (85–87). Despite the lack of available reliable CPAP devices, a systematic review of 21 observational quasi randomized and observational studies using improvised CPAP devices concluded that the introduction of CPAP improved neonatal survival (81). Pooled data from four of the observational studies showed 66% reduction in in-hospital mortality among preterm neonates following introduction of CPAP (odds ratio 0.34, 95% confidence interval 0.14–0.82) (81).

In 2020, UNICEF published a target product profile to align CPAP innovators and other stakeholders to the most important performance and operational characteristics, as well as target pricing to aid in the development of effective and safe CPAP devices that can be scaled (88). Excerpts from this profile are shown in Table 1. Some technical characteristics for optimal CPAP devices include the ability to produce CPAP pressure between 5 and 8 cm H_2_O, provide humidification, blend oxygen, and have flow capacity of 0–10 L/min. Table 2 compares the characteristics of different categories of CPAP devices available in LMIC to the highest cost devices which fulfill the requirement of UNICEF's CPAP target product profile (89).

**Table 1:** Features from the UNICEF target product profile for neonatal continuous positive airway pressure for use in low- and middle-income countries (88).

**Characteristic**	**Optimal**	**Minimal**
**Technical characteristics**		
Flow driver	Integrated (on-board air compressor)	
Oxygen flow capacity	0–10 LPM	
Pressure	5–8 cm H_2_O	
Total blended flow	0–10 LPM	
Humidification	Heated humidification	None
Alarms	Audio and visual	Audio power
**Purchasing**		
Accessories	Non-proprietary	Proprietary
Consumables	Reusable	Available
Instrument pricing (without shipping costs)	<US$1,000	<US$2,000
**Utility requirements**		
Power source	Mains with battery backup	Mains
Battery	Rechargeable	None
Voltage	Matches that available in purchasing country	

**Table 2:** Comparison of features and cost of CPAP device categories in use in LMIC.

	**Improvised circuits**	**Low-cost**	**Medium cost**	**High cost**
Required flow source	Oxygen			Pressurized oxygen and air
Oxygen blending	No	Yes		
Humidification	Passive (bubble bottle) and entrained ambient humidification			Heated humidified air
Patient interface	Nasal cannula	Hudson prong or RAM cannula	Proprietary prongs, RAM cannula or Hudson prong	Proprietary prongs/mask, RAM cannula or Hudson prong
Tubing and interface resistance	High (tubing and nasal prongs)	May include high resistance components	Low	Low
Electricity requirement	None for device. Is needed for oxygen concentrator if used and pulse oximeter.		Required	
Consumables	Single use cannula	Single and multi-use components	Single and multi-use components	Single use components
Cost (USD)	$1–4	$20– $800	$1,000–2,000	$3,000–6,000+
Example devices	WHO (83)	Vayu (90)	Pumani (91)	Fisher-Paykel (89)
		PATH (77)	Diamedica (92)	Dolphin (70)
			Polite (93)	Phoenix (71)

*Updated from reference (72)*.

The only published LMIC randomized trial of CPAP among neonates was conducted in Tanzania, in which 48 preterm infants with birth weight >1,000 g with RDS were randomized to receive CPAP *via* the Pumani device (25 subjects) or oxygen (23 subjects). The study found that survival to hospital discharge in the CPAP and oxygen groups respectively was not statistically significant [68 vs. 47.8%, crude OR 2.3 (95% CI 0.72–7.49)]. However, in the per-protocol analysis a more significant number of subjects in the CPAP group survived to hospital discharge 77.2 vs. 47.8% (crude OR 3.7, 95% CI 1.02–13.47). The study reported no pneumothoraxes, but that bleeding from the nose was more common in the CPAP group (94).

Other available evidence on the benefits of CPAP from LRS include three trials conducted in children who were between 1 month to 5 years of age with diagnoses other than RDS. The first trial conducted in Ghana was a cross over trial between two secondary hospitals and included over 2,000 subjects with clinical signs of respiratory distress. The primary outcome measure was all-cause mortality at 2 weeks after enrollment. The study found that 3% (26/1,021) patients in the CPAP group, and 4% (44/1,160) patients in the control group died [relative risk (RR) of mortality 0.67, 95% CI 0.42–1.08; *p* = 0.11] (95). The second was conducted in Bangladesh and randomized children with a diagnosis of severe pneumonia and hypoxemia to receive oxygen therapy by either bubble CPAP, standard low-flow nasal cannula (2 L/min), or high-flow nasal cannula (2 L/kg per min up to the maximum of 12 L/min). Significantly fewer children in the bubble CPAP group had treatment failure than in the low-flow oxygen therapy group (relative risk 0.27, 99.7% CI 0.07–0.99; *p* = 0.0026) (96). The third trial was conducted in Malawi, and 323 children were randomly assigned to oxygen and 321 to bCPAP. The results showed that CPAP was associated with increased risk of mortality compared with oxygen therapy, 53 (17%) of 321 vs. 35 (11%) of 323 (relative risk 1.52; 95% CI 1.02–2.27; *p* = 0.036) (97).

The lack of standard equipment and high-quality trials have limited access to CPAP among preterm neonates with RDS in LRS. There are also practical barriers to the widespread use of CPAP in LMICs. In a systematic study on facilitators and barriers to implementation of CPAP in LMICs, the authors reported staff shortage and high staff turnover limited the uptake and use of CPAP (76). The study also reported that parents were resistant to the use of CPAP because of local beliefs that oxygen use led to poor outcomes (76).

#### Oxygen blending

Supplemental oxygen is a key RDS treatment modality whether used through nasal cannula, CPAP or mechanical ventilation (98). However, oxygen use among neonates in particular can have deleterious effects and current evidence is that titration of fraction of inspired oxygen to achieve saturations as measured by pulse oximetry between 90 and 95% provides the optimal balance between the therapeutic benefits of oxygen and the risk of oxygen toxicity (45). When used in excess, oxygen can cause oxidative injury to a premature baby's lungs, eyes and brain (99). Use of 100% oxygen with premature infants is a major risk factor for the development of retinopathy of prematurity (ROP) which can lead to visual impairment and even blindness among survivors (45). This is especially important in CPAP and mechanical ventilation where all of the baby's inspired gas is from the respiratory circuit and there is no entrainment of room air around a nasal interface or mask. Most low resource facilities treating newborns with CPAP use 100% oxygen because they do not have a source of compressed air to blend with oxygen (73, 74, 100). The well-intentioned use of oxygen therapy to save preterm newborn lives in LRS could lead to an epidemic of ROP-related blindness in sub-Saharan Africa, as already suggested in Latin America, South Africa, India and China (101–104). Methods to ensure safe use of oxygen, with blending of air to optimize the fraction of inspired oxygen are urgently needed (66). Recent WHO guidelines for the care of small and sick newborns recommend retinal exams for preterm infants to detect ROP and efforts to scale up this screening are underway (45, 105). Unfortunately, ophthalmologists trained in these exams are sparsely distributed (106).

Several options exist for the provision of blended oxygen to newborns with varying cost and availability (Table 3). High resource settings and some tertiary facilities in LRS use precision oxygen blenders; however, these are expensive and require high pressure sources of air and oxygen flow. Some medium cost commercialized CPAP devices designed for lower resource settings include on-board air compressors. When used in combination with an oxygen source, these devices can provide flow with oxygen concentrations ranging from 21 to 100% when available (92–94).

**Table 3:** Oxygen blending modalities for CPAP in low resource settings.

	**No blending**	**Blending** ***via*** **ambient air entrainment device**	**Blending** ***via*** **air compressor in CPAP device**	**Blending with high pressure (wall) sources**
Range of percent oxygen	100%	30–100%	21–100%	21–100%
Availability in low resource facilities	Most frequently used	Not yet commercially available	Increasingly in use	Generally limited to tertiary facilities
Components Required	– Oxygen tank or concentrator	– Oxygen tank or concentrator	Medium cost CPAP device or stand-alone air compressor Oxygen tank or concentrator	High precision blender High pressure air and oxygen sources
Relative Cost	$	$$	$$$	$$$$$
Example devices	WHO (83)	PATH (77) Vayu (107) Minnesota (108)	Pumani (94) Diamedica (92) Polite (93)	Precision medical (109) Bio-med devices (110)

To address the remaining gap in safe oxygen therapy, low-cost modalities to blend oxygen and air are in development. One type of device entrains room air into a flow of oxygen *via* the Bernoulli principle and therefore does not require compressed air (77, 107, 108). Initial studies of these devices are in progress and show promise in their efficacy, portability, cost, and usability. This entrained air mechanism, however, is limited to providing fraction of inspired oxygen in pressurized flow of less than ~30% because of the pressure drop associated with increasing entrainment of room air (107).

An important consideration for provision of blended oxygen is the increased importance it places on pulse oximetry and measurement of patient saturations to guide blending at the bedside. Pulse oximetry is included as a recommendation in the WHO standards for care of the small newborns and is best used continuously to allow frequent detection of low or high saturations and resultant adjustment in oxygen concentration (45). However, oximetry devices are expensive and remain a limitation for safe provision of CPAP and oxygen (74, 111).

#### Methylxanthines

In addition to RDS, apnea of prematurity (AOP) commonly affects premature infants. It is defined as cessation of breathing with hypoxia and bradycardia that last more than 15 s (112). The severity and frequency of AOP are inversely related to the degree of prematurity (112, 113). Methylxanthines—aminophylline, theophylline, and caffeine citrate (caffeine)—are the mainstay pharmacologic treatments for AOP used adjunctively with positive pressure ventilation (114). The pharmacological effects of methylxanthine include (i) stimulation of the respiratory center in the medulla; (ii) increased sensitivity to carbon dioxide; (iii) increased skeletal muscle tone; (iv) enhanced diaphragmatic contractility; (v) increased minute ventilation; (vi) increased metabolic rate; and (vii) increased oxygen consumption (115–117). Caffeine and aminophylline are equivalent in reducing events of AOP (118). However, caffeine has a wider therapeutic index, longer half-life that allows once-daily administration, does not require drug-level monitoring, and has a better side effect profile—causing less tachycardia and feeding intolerance (118, 119). Furthermore, compared to placebo, caffeine shortens ventilator days, reduces the risk of developing bronchopulmonary dysplasia, improves neurodevelopment at 18–24 months, and is cost-effective (120–123). Furthermore, theophylline therapy has been associated with seizures and hypokalemia in neonates (124).

Caffeine for AOP treatment first appeared in the 2009 WHO essential drug list (125). Despite this, the use of caffeine to treat preterm babies in low resource settings has not achieved scale (126). In a survey of 55 clinicians from 13 countries in sub-Saharan Africa, only six countries used caffeine, and often inconsistently (126). In a review of 11 studies on caffeine for premature infants, the only studies from LMICs were from India (127). The reasons for the unavailability of caffeine in SSA are complex, including high drug prices, stock outs, and the drugs is not obtainable for purchase in some countries (126). Additionally, there may be less demand for caffeine as knowledge of greater safety over aminophylline may not be pervasive. Furthermore, the patient population where the benefits of methylxanthines are greatest (very-to-extreme preterm infants), have very poor survival in LMICs. The availability of overall care for these patients varies greatly between these low resource sites and that in HIC where the caffeine trials were conducted (120, 128).

#### Surfactant

Exogenous surfactant replacement therapy is the definitive pharmacologic treatment for RDS (129). Used adjunctively with invasive or non-invasive ventilation, the use of surfactant reduces RDS specific neonatal mortality (130). WHO recommends that small and sick neonates be assessed for surfactant deficiency, and treatment provided within 2 h of birth (45). Exogenous surfactant preparations include synthetic surfactants and natural surfactants derived from animal sources. Early trials suggested that natural surfactants are more efficacious than synthetic preparations, with the benefit attributed to the proteins in natural preparations (131). However, synthetic surfactants can be produced at lower cost, and newer synthetic surfactants are being developed and evaluated, showing promise for their equivalency to natural surfactants (132, 133).

Surfactant use is limited in low resource settings; particularly, sub-Saharan Africa (74, 134). In a systematic review on the use of surfactant in LMICs, of 38 relevant studies, none were from Sub-Saharan Africa (SSA) (135). In a survey involving respondents from 49 African countries, surfactant was available in 33% and 39% of the most well-equipped public and private hospitals, respectively (74). Potential explanations for the limited use of surfactant in LMICs includes unavailability, cost, and the perception that surfactant therapy must occur in conjunction with mechanical ventilation (MV) which may not be available or feasible (74). In the survey of African respondents, the cost of a vial of surfactant varied from <US$200 to over US$500.

Traditional surfactant replacement therapy involves instillation *via* endotracheal tube, which requires significant skill on the part of the provider, and adjunctive use of MV. However, given the association of MV with adverse outcomes (136–138), in current neonatal practice, clinicians strive to limit or avoid mechanical ventilator use. This change in practice has led to innovative ways to administer exogenous surfactant in minimally invasive ways. Examples of these techniques include dosing surfactant *via* a thin catheter, a laryngeal mask airway (LMA), or using nebulized or aerosolized surfactant. LMAs, of note, are generally designed for use among neonates >2,000 g, and studies on surfactant instilled *via* LMA have not included preterm newborns <1,250 g (139).

**Figure 3:**
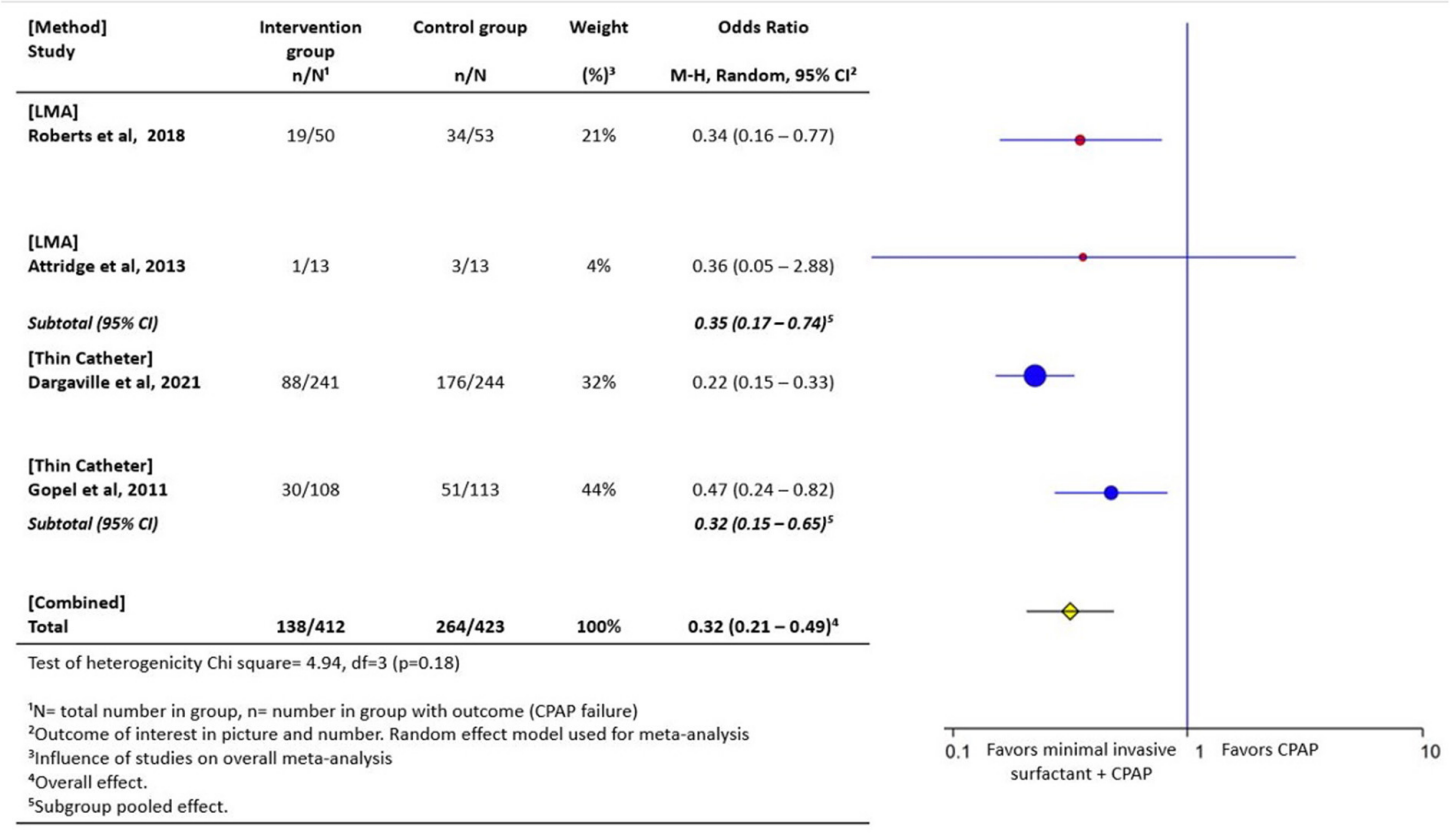
Forest plot of comparison of CPAP plus minimally invasive surfactant administration *via* thin catheter or laryngeal mask airway (LMA) vs. CPAP only. The outcome of interest was CPAP failure as determined by need for intubation. These minimally invasive techniques are associated with a 47% risk reduction in CPAP failure (142–144, 150).

Evidence from high-income settings indicates that instillation of surfactant through a thin catheter (140, 141) or LMA method (142, 143) can prevent the need for intubation when compared to treatment with continuous positive airway pressure alone. When pooled in a meta-analysis, these minimally invasive techniques (administration *via* LMA or thin catheter) are associated with a 47% risk reduction in CPAP failure, when compared with CPAP alone (141–144) (Figure 3). Surfactant instillation *via* thin catheter is included in the 2019 European consensus guidelines on the management of respiratory distress syndrome (145, 146). Data from the German Neonatal Network indicated that over 50% of surfactant replacement therapy occurs with the thin catheter method (147). Results on the efficacy and feasibility of nebulized surfactant is emerging (148). In a recent randomized controlled trial, nebulized surfactant did not differ from CPAP alone in preventing CPAP failure, however with advances in nebulization/aerosolization devices and surfactant formulations, this modality may have promise (149).

Data from a survey conducted among 49 African countries on the availability of neonatal respiratory care showed that 11% (4/35) of NICUs capable of providing surfactant replacement therapy did so with the thin catheter method (74). There are no randomized trials originating from a LMIC on the use of minimally invasive surfactant administration. The only available study from Sub Saharan Africa is an observational study from Asaba, Nigeria that compared mortality among preterm infants with respiratory distress treated with CPAP who were administered surfactant *via* thin catheter compared with those who did not receive surfactant. Eligibility included parent's ability to pay for the surfactant, resulting in only 25% (*n* = 51) of neonates to be able to receive the medication. The study reported a significant reduction in mortality with surfactant administration only among neonates with birth weight below 28 weeks and 1 kg birthweight [50% (13/26) vs. 65% (35/54)] (151). Further studies on less invasive strategies for administration of exogenous surfactant in LRS are urgently needed.

#### Mechanical ventilation

Oxygen therapy with CPAP for RDS is the most prevalent form of respiratory support in LMIC (74, 100). However, in a systematic review that included eight observational studies from LMIC on the use of CPAP and enrolling patients of varied degrees of prematurity, 20–40% of infants with RDS failed CPAP treatment in the absence of surfactant therapy (81). These patients failing CPAP require surfactant administration and mechanical ventilation (MV) for improved survival. In a survey on available respiratory support modalities for RDS from Africa, of the 49 countries with at least one respondent, only 49% of the most well-equipped government hospitals and 59% of the most well-equipped private hospitals located in capital cities used MV (74). A survey from Nigeria suggests sparse availability and lack of capacity to use MV devices where available (152, 153). We speculate that the findings of this survey can be generalized to other LMICs. Most global recommendations and predictive models of interventions to improve prematurity related mortality in LMICs do not include MV, only CPAP and there is a need to elucidate the residual mortality from RDS which could be averted with MV (45, 154).

The cost of MV and lack of capacity to use MV are deterrents to its use (74). MV is not a standalone intervention; medications like surfactant, caffeine, and antenatal steroids improve outcomes of neonates managed on MV. Furthermore, the use of blended oxygen, oximetry, arterial blood gas, mobile chest x-rays, respiratory therapists, neonatal nurses, biomedical technicians, and pharmacologic support are critical for safe and effective use of MV but are limited in LMIC. MV is currently not taught and not incorporated into undergraduate training of medical and nursing schools in several LMICs (74, 155). Furthermore, postgraduate clinical exposure and training on MV is limited; hence knowledge of MV among the essential workforce is deficient. Data on MV's safety and efficacy in LMIC is currently lacking; this area needs further exploration.

During the COVID-19 pandemic, the lack of, and need for MV generated awareness of its necessity in LMICs among patient populations outside of the neonatal period (156–158). The pandemic also sparked the production of more basic, compact, and affordable MV devices by academic institutions and industry (159–161). The increased access to, and availability of MV in some centers may increase its use in newborn respiratory support which should be done with caution.

### Fluid and nutrition

Adequate nutrition is critical for the continued growth and development of the premature respiratory system. Premature infants are at risk of insensitive water losses and nutritional failure by virtue of their dermal and intestinal immaturity. Liberal or excessive fluid administration is associated with poor respiratory outcomes in preterm infants (162), and optimal early nutrition is correlated with a better pulmonary outcome (163). Hence careful consideration of fluid management, and optimizing nutrition in premature infants is essential to RDS management.

## Discussion

To achieve the sustainable developmental goals targets, small and sick newborns need high-quality inpatient care at the right time and in the right place (8). Transformative RDS therapies, which are standard of care in high resource settings (HRS), significantly reduce prematurity-related mortality. However, 40–60 years after these interventions were proven effective, about 2.4 million small and sick newborns born in LMICs will die this year because of lack of access. The resource limitations in LMICs that affect medication and equipment availability and the ancillary care required to reap their benefits are poor or non-existent. Consequently, our knowledge on the impact of these RDS-specific interventions in LRS is limited. Important questions that remain include:

1. how women at risk of preterm labor in rural communities can be treated safely with ACS and have the desired pregnancy outcome?
2. how can low-cost CPAP devices be optimized to be as effective and safe as the gold standard devices?
3. how can providers learn and maintain direct laryngoscopy skills and the complexities of MV?
4. how can the cost of essential medications like surfactant, caffeine, and devices like CPAP and MV be made more affordable, so they are accessible to providers in LMICs?

To bridge this knowledge gap, thoughtful research designs are required. Research needs to focus equally on implementation as well as evidence generation, taking into context the resource limitations of the clinical setting (164). These type of research studies will allow accurate determination of the extent to which timely and effective neonatal transport, thermoregulation, nutritional support, hypoglycemia management, infection control, and provider-to-patient ratios affect the benefits of these evidence-based RDS-specific therapies. Also, the use of telemedicine proved beneficial during the COVID-19 pandemic where face to face physician patient interaction was limited. Indeed, telemedicine has been used to provide simulation training effectively and feasibly for neonatal resuscitation skills (165, 166). Opportunities to leverage telemedicine in overcoming some of the highlighted barriers is an area for further exploration.

Most global recommendations and predictive models of interventions to improve prematurity-related mortality in LMICs indicate that the greatest benefit will only be achieved with a comprehensive approach to implementing these evidence-based interventions (45, 154). Critical to the impact of any RDS-specific interventions are the core elements of neonatal care including thermoregulation, KMC, safe and adequate transportation to facilities capable of providing high-quality care, infection management, fluid and nutritional support, and workforce availability and capacity. Meeting the ENAP coverage target for care of small and sick newborns in facilities that can provide basic RDS-specific management like optimal resuscitation and CPAP in 80% districts will require considerable investment. The target will be more attainable with further development of low cost, safe and reliable RDS-specific drugs and devices; broader availability consumable parts, standardization of training, guidelines for optimal and safe use, in addition to adequate staffing to provide this level of care (7, 75, 167). To accelerate the development of solutions essential to make the global coverage target feasible, governments may consider providing incentives for local and international biomedical companies to enable them to safely and effectively produce and market their products locally. These incentives could include tax exemptions, streamlined in-country product registration and evaluation by regulatory bodies.

## Conclusion

Respiratory distress syndrome is a major driver of prematurity related neonatal mortality. High quality trials conducted in HRS have shown interventions to be effective in reducing the RDS specific neonatal mortality. However, these standard of care interventions in HRS have sparse and sporadic coverage in LRS. When tested in LRS these interventions have not consistently had the same beneficial effect, likely due to the lack of critical ancillary services and core neonatal care practices. To achieve a neonatal mortality rate as low as 12 per 1,000 live births in year 2030 (8), guided by high quality implementation and effectiveness research, considerable scale up of RDS-specific interventions bundled with core neonatal care practices are needed.

## Author contributions

OE, MB, and AH conceptualized the review. Initial draft by OE, IO, and AH with revisions by MB. All authors contributed to the article and approved the submitted version.

## Conflict of interest

The authors declare that the research was conducted in the absence of any commercial or financial relationships that could be construed as a potential conflict of interest. The handling Editor declared a shared committee with one of the authors MB.

## Publisher's note

All claims expressed in this article are solely those of the authors and do not necessarily represent those of their affiliated organizations, or those of the publisher, the editors and the reviewers. Any product that may be evaluated in this article, or claim that may be made by its manufacturer, is not guaranteed or endorsed by the publisher.
